# In response to Shimizu et al. (2026)

**DOI:** 10.1002/acm2.70613

**Published:** 2026-05-24

**Authors:** Hayato Tsuno, Ruan Sasaki, Koji Sasaki, Kohei Nishi, Tae Ushikawa, Daisaku Goto, Yasuhiro Kawashima, Ririko Kosugi

**Affiliations:** ^1^ Gunma Prefectural College of Health Sciences Maebshi Gunma Japan; ^2^ Japanese Red Cross Maebashi Hospital Maebshi Gunma Japan; ^3^ National Hospital Organization Sendai Medical Center Miyagi Japan; ^4^ Graduate School of Radiological Technology Gunma Prefectural College of Health Sciences Maebshi Gunma Japan; ^5^ JR Tokyo General Hospital Tokyo Japan; ^6^ Hamamatsu University School of Medicine Shizuoka Japan

## 1 POINTS ALREADY DESCRIBED AS STUDY LIMITATIONS

The characteristics of the proposed current source raised in the comment, including load dependence and the influence of internal circuitry, have already been recognized and explicitly described as limitations of this study.

In the present work, we clearly defined the applicable scope and underlying assumptions of the proposed method. Therefore, we consider that many of the points raised in the comment correspond to issues that have already been acknowledged and discussed in the manuscript.

## 2 ON THE EMPIRICAL BASIS OF THE COMMENT

Importantly, many of the concerns raised in the comment are not supported by specific experimental data or quantitative evaluation, but rather remain at the level of general theoretical possibilities based on circuit analysis.

In contrast, our study experimentally evaluated the performance of the proposed method using electrometers through comparative measurements. We demonstrated that the proposed *in‐house second‐channel calibration* approach achieves repeatability and uncertainty comparable to those of conventional methods. In addition, separate experiments comparing the proposed method with current sources equipped with servo circuits showed consistent results.

Furthermore, previous studies have emphasized the importance of long‐term stability assessment in electrometer quality assurance and have shown that continuous in‐house measurements can effectively detect temporal variations.

In this context, the concerns raised in the comment remain hypothetical and lack direct experimental validation.

## 3 DISTINCTION FROM TRACEABLE CALIBRATION

In discussing this topic, it is essential to distinguish between traceable calibration and in‐house constancy checks (intercomparison), as they serve fundamentally different purposes.

External calibration ensures metrological traceability to national standards and guarantees the absolute accuracy of measurements. In contrast, the primary purpose of in‐house procedures is to monitor temporal changes (drift) in instrument sensitivity using a transfer standard, rather than to determine absolute values.

As described in the manuscript, the current values in our method are treated as relative within this framework.

## 4 COMPLEMENTARY ROLE IN QUALITY ASSURANCE

These two approaches are not mutually exclusive but are complementary. A practical quality assurance strategy combines high‐accuracy external calibration (e.g., every few years) with more frequent in‐house verification (e.g., semiannually), enabling early detection of unexpected failures or degradation such as insulation breakdown or component drift.

From this perspective, the requirement for the current source used in in‐house verification is not necessarily high absolute accuracy, but rather high repeatability and stability under consistent conditions.

Under such conditions, the proposed *battery‐powered current source* can serve as a practical and effective tool for detecting relative changes in electrometer response.

## 5 CLINICAL SIGNIFICANCE

In recent years, there has been a growing emphasis on *in‐situ calibration and verification* in the fields of industrial and measurement science, where instruments are evaluated without removal from their operational environment.

Our study represents an innovative attempt to extend electrometer management in clinical practice from reliance solely on external calibration services to an engineering‐based in‐house quality assurance framework.

This approach has the potential to avoid situations in which patient‐specific QA cannot be performed while instruments are sent for external calibration, and to enable early detection of errors within the clinical facility, thereby contributing to safer radiotherapy practices. Rather than viewing these approaches as mutually exclusive, the authors hope that science can provide patients with a better future.

## 6 CONCLUDING REMARKS

In summary, the evaluation of the proposed method should be based not only on theoretical considerations but also on quantitative experimental evidence.

If there are concerns about the validity of this approach, we believe that specific measurement data and an uncertainty analysis should support them.

Sincerely yours,

## CONFLICT OF INTEREST STATEMENT

The authors have nothing to report.

